# Tailor-made composite functions as tools in model choice: the case of sigmoidal *vs* bi-linear growth profiles

**DOI:** 10.1186/1746-4811-2-11

**Published:** 2006-06-02

**Authors:** Winfried S Peters, Tobias I Baskin

**Affiliations:** 1 Indiana University/Purdue University, Department of Biology, 2101 E Coliseum Blvd, Fort Wayne IN 46805-1499, USA; 2 Biology Department, University of Massachusetts, 611 N Pleasant St, Amherst MA 01003, USA

## Abstract

**Background:**

Roots are the classical model system to study the organization and dynamics of organ growth zones. Profiles of the velocity of root elements relative to the apex have generally been considered to be sigmoidal. However, recent high-resolution measurements have yielded bi-linear profiles, suggesting that sigmoidal profiles may be artifacts caused by insufficient spatio-temporal resolution. The decision whether an empirical velocity profile follows a sigmoidal or bi-linear distribution has consequences for the interpretation of the underlying biological processes. However, distinguishing between sigmoidal and bi-linear curves is notoriously problematic. A mathematical function that can describe both types of curve equally well would allow them to be distinguished by automated curve-fitting.

**Results:**

On the basis of the mathematical requirements defined, we created a composite function and tested it by fitting it to sigmoidal and bi-linear models with different noise levels (Monte-Carlo datasets) and to three experimental datasets from roots of *Gypsophila elegans*, *Aurinia saxatilis*, and *Arabidopsis thaliana*. Fits of the function proved robust with respect to noise and yielded statistically sound results if care was taken to identify reasonable initial coefficient values to start the automated fitting procedure. Descriptions of experimental datasets were significantly better than those provided by the Richards function, the most flexible of the classical growth equations, even in cases in which the data followed a smooth sigmoidal distribution.

**Conclusion:**

Fits of the composite function introduced here provide an independent criterion for distinguishing sigmoidal and bi-linear growth profiles, but without forcing a dichotomous decision, as intermediate solutions are possible. Our function thus facilitates an unbiased, multiple-working hypothesis approach. While our discussion focusses on kinematic growth analysis, this and similar tailor-made functions will be useful tools wherever models of steadily or abruptly changing dependencies between empirical parameters are to be compared.

## Background

Kinematic growth analysis aims at the quantitative description of spatial growth patterns to provide a basis for the study of developmental mechanisms [1,2]. As the term *kinematic *indicates, this approach focuses on the movement of parts of a growing organ relative to each other. The concepts for kinematic growth analysis were laid out half-a-century ago for the root, which was assumed for simplicity to grow uni-directionally (*i.e*. pure elongation or axial growth) [3-5]. The assumption of pure elongation keeps the mathematics manageable. Consequently, the vast majority of studies applying kinematic growth analysis to physiological problems have focused on pure elongation in suitable organs such as roots [6-8] and grass leaves [9-11].

The key parameter is the velocity field, the spatial distribution of the velocities at which these displacements occur. For uni-directional expansion, the velocity field reduces to a velocity profile. The derivative of the velocity profile has often been referred to as the *relative elemental elongation rate*. This rate is *relative *because it is a measure of growth that is independent of the size of the growing entity, with the dimension of reciprocal time, and it is *elemental *because it represents a calculus-based description of infinitesimal elements of tissue [2]. However, an elemental rate is by definition a relative rate. To avoid this redundancy, we drop the *relative *and use simply *elemental growth *(or *elongation*) *rate *to refer to the spatial derivative of a velocity profile. This usage also helps to avoid confusion between an elemental growth rate, which describes motion within a spatial system of reference, and the relative growth rate, a well-established concept in classical growth analysis which describes changes in size over time. Here we are concerned primarily with the former type of rate, although the analytic tool we introduce might prove useful in classical growth analysis as well.

**Figure 1:**
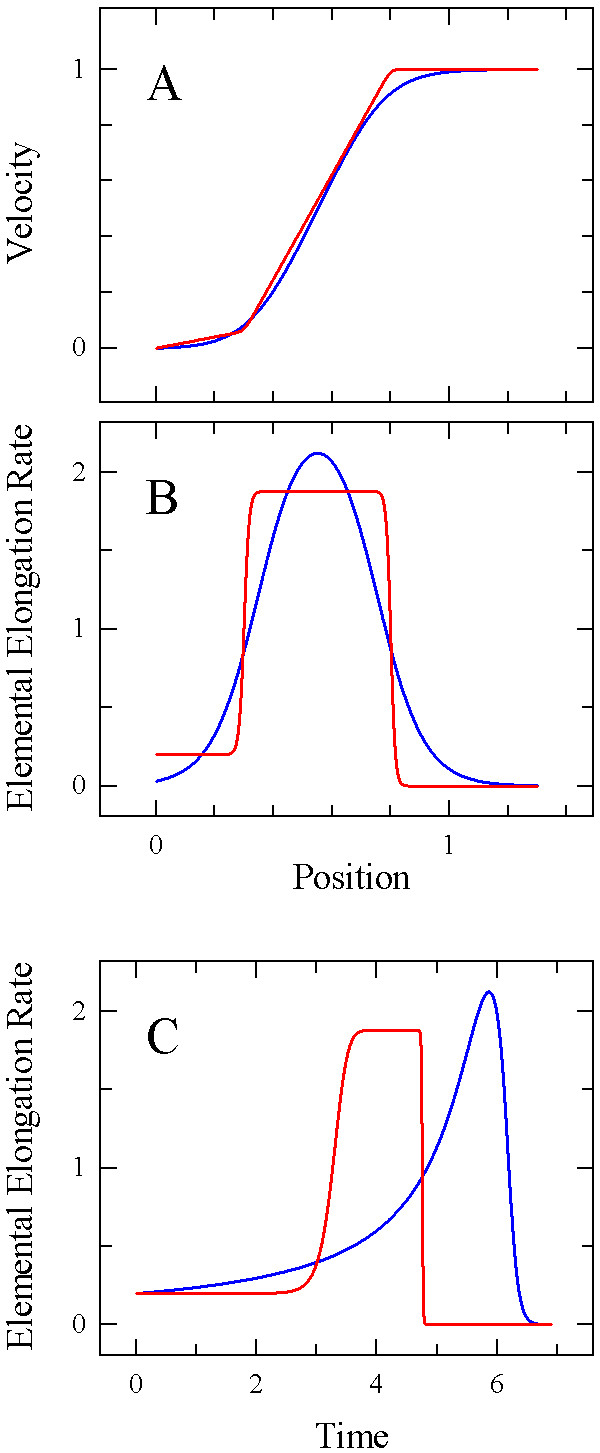
**Schematic comparison of sigmoidal and bi-linear growth profiles**. (A) Sigmoidal (blue) and bi-linear (red) model profiles of velocity; zero on the abscissa corresponds to the root tip. (B) Derivatives of the velocity profiles in (A) with respect to position, yielding profiles of elemental elongation rates. The distinct character of the two growth profiles becomes more evident in (B) than in (A). (C) Time-courses of elemental elongation rates calculated from the velocity profiles (A). Time 0 was chosen to correspond to the position at which the two rate profiles cross over for the first time (position 0.155 in (B)). The curves show the elemental elongation rate experienced over time by a point initially located at that position on the root.

Velocity profiles along growing root tips and leaves have been widely reported to be sigmoidal; concomitantly, the corresponding elemental growth rate profiles reported were bell-shaped, with a single, smooth peak. Recently, for the root, determination of velocity profiles at greatly increased temporal and spatial resolution has produced distributions that appeared to be bi-linear ([12]; for a comparison of sigmoidal and bi-linear velocity profiles, see Fig. 1). Accordingly, the corresponding elemental growth rate profiles had "step-stool"-shapes, showing two relatively stable plateaus (Fig. 1B). Intriguingly, profiles along growth zones of anatomic parameters such as cell length, which under steady state conditions and in the absence of cell division are geometrically similar to the corresponding velocity profiles [13], have occasionally been interpreted as being bi-linear [14-16]. The recent report provides experimental support to the suspicion that sigmoidal velocity profiles might, at least in some cases, be artifacts resulting from measurement error, averaging, or insufficient spatial or temporal resolution [12].

The distinction between sigmoidal and bi-linear velocity profiles is important biologically. As a root cell traverses a sigmoidal growth zone, it increases and then decreases its elongation rate steadily and smoothly from one end of the zone to the other. In contrast, as a cell traverses a bi-linear growth zone, it elongates at one steady rate for part of the zone, rapidly increases to a new rate for the rest of the growth zone, and then stops (Fig. 1C). In the sigmoidal case, regulation of elongation rate is expected to be continuously variable whereas in the bi-linear case, the regulation should establish two distinct rates of elongation as well as the positions where the transitions occur. In reality, no growth zone will be exactly bi-linear because elemental growth rates cannot be perfectly constant and change instantaneously in a mathematical sense. It is an open question whether some growth zones are purely sigmoidal but it appears possible that all or most root growth zones have a mixture of sigmoidal and bi-linear characteristics.

The analytical power of kinematic growth analysis rests on the fact that knowledge of the velocity field enables one not only to calculate the local rates of deposition of any parameter of interest, whether it be cells, cell wall material, solutes, or water, but also to compute time-courses of these and other parameters as experienced by cells that traverse the growth zone [17-20]. To exploit this analytical power in full, the profiles of velocity and elongation rate need to be rendered as continuous functions. For this reason, kinematic growth analysis is associated with curve-fitting, where some function (or group of functions) is fitted to the raw velocity data.

Because at present there is no mechanistic model for the regulation of the velocity field within a growth zone, the choice of a function to fit is arbitrary. As velocity profiles generally resemble sigmoid curves, authors have applied sigmoid functions previously established in classical growth analysis (such as the Gompertz or Richards functions; [21]) as well as versions developed specifically for kinematic growth analysis [22,23]. However, such functions will smooth out regions of the profile whose behaviour departs from the sigmoid. Alternatively, one may use a piecewise approach where polynomials are fit to small, overlapping subsets of the data [2,7]. This approach excels at capturing local behaviour but nevertheless smoothes abrupt transitions [12] and can be difficult to apply to noisy data. Alternatively, van der Weele et al. [12] fitted linear regression lines to the velocity data, but this approach yields profiles with discontinuous derivatives and allows no possibility for any sigmoid character. Describing stem elongation over time, Fisher et al. [24] introduced a 3-phasic equation which avoided discontinuous derivatives, but which was based on the assumption that a linear growth phase did in fact exist.

To analyze velocity profiles without forcing them to be either sigmoids or to contain a straight line, we formulated a function which describes sigmoidal and bi-linear profiles equally well. The result of fitting such a function provides an independent criterion to distinguish between the two types of profile, as well as an estimate of the transition point positions and their degree of abruptness. The function is a composite of terms chosen to satisfy defined requirements. We suggest that this kind of tailor-made function created for specified purposes can be a useful tool for solving various analytical problems in growth research.

## Theory

### Requirements of the function

The desired function must demonstrate the following properties of velocity profiles:

• At x = 0, y = 0. That is, velocity is defined to be zero at the point of reference. Note that this definition inverts the intuitive frame of reference. With respect to the plant's environment, the root tip has the maximal velocity and velocity falls to zero at a point that defines the end of the growth zone. In contrast, when the root tip is chosen as the reference point, velocity is zero at the tip and rises to a maximum at the point defining the end of growth zone. This inversion of the reference frame provides a host of mathematical advantages and is ubiquitously employed in kinematic analyses [1].

• It must be able to describe a series of three intervals of linear relationship between x and y: the slopes of the first and second intervals will be positive with the second slope greater than the first, and the slope of the third interval will usually be zero. The transitions between linear domains must be continuous.

• It also must be able to describe a sigmoidal relation between x and y. In other words, the function should be able to mimic conventional growth equations up to practical identity.

As we will see, a function that satisfies these requirements can be assembled using six coefficients. Two are needed to determine the non-zero slopes of the first two linear domains; we call these linear factors *b*_1 _and *b*_2_, respectively, with the subscript indicating which of the two linear domains the coefficient controls. Two coefficients are required to determine the positions (*c*_1 _and *c*_2_) of the two transitions, and the final pair of coefficients (*d*_1 _and *d*_2_) determine the extent of the transitions. The extent of the transitions defines the linear versus sigmoidal character of the profile: the more extensive the transitions relative to the overall length of the profile, the more sigmoid-like the curve. In the following, we describe the function by breaking it down into its component building blocks.

### Assembly of the step-stool function

The basic element of the function is an exponential term which can be used to create a smooth transition between two linear domains. Let us consider this term first in a form that resembles the so-called "expolinear" expression used in classical growth analysis [25,26]:



where *b*, *c*, and *d *are constants and *x *is the variable. When *c *>> exp(*b d x*), then *y *reduces to the constant (ln *c*)/*d*, indicating that at small values of *x*, there is a linear domain that has zero slope. On the other hand, when *c *<< exp(*b d x*), then *y *approaches (*b x*). Therefore, the function defines a transition between a line of zero slope at small *x *and one of slope *b *at large *x*. The transition is characterized by its location and width, which are determined by the coefficients *c *and *d*.

However, with equation (1), there is no direct correspondence between *c *or *d *and the location of the transition. Such a direct correspondence is desirable, though, because it would facilitate the process of initial value estimation, important for practical curve-fitting. Looking again at equation (1), we note that the center of the transition zone is located at the *x *value at which *c *= exp(*b d x*). Thus, if we replace the coefficient *c *in equation (1) by the expression exp(*b c*_1_*d*), the new coefficient *c*_1 _will have the value of *x *at the center of the transition. We now rewrite equation (1) using the conventions for coefficient identification defined above, and arrive at



**Figure 2:**
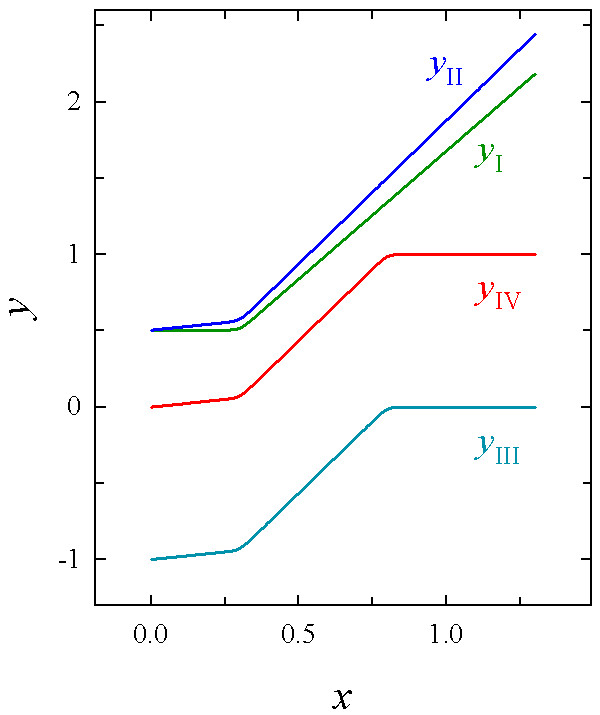
**Assembly of the step-stool function**. Four consecutive steps (*y*_I _to *y*_IV_) in the assembly of a continuous function capable of describing sigmoidal and bi-linear growth profiles equally well. See Theory section for details. Coefficients are *b*_1 _= 0.2, *b*_2 _= 1.69, *c*_1 _= 0.3, *c*_2 _= 0.8, *d*_1 _= *d*_2 _= 50.

A sample *y*_I _with rather narrow transition zone centered on *x *= 0.3 is shown in Fig. 2.

Obviously, the assumption that the first linear domain has zero slope does not necessarily hold for real velocity profiles. To allow for non-zero slopes, a linear term, *b*_1_*x*, has to be added to *y*_I_:



where, for simplification, we define:

*K *= ln (exp [*b*_2 _*c*_1 _*d*_1_] + exp [*b*_2 _*d*_1 _*x*])     (4)

In *y*_II_, the slope of the first linear domain is *b*_1_, while that of the second equals *b*_1 _+ *b*_2 _(Fig. 2). As we will use it later in forcing the complete function to attain the value 0 at *x *= 0, we note that the value of *y*_II _at *x *= 0 is



To create a second transition between the second linear domain and a third one with zero slope (which corresponds to the non-growing parts of the root proximal of the growth zone), we employ the same tools. Taking another look at equation (2), we see that the variable *x *appears only in the term *b*_2_*d*_1_*x *in the second exponential expression, and that the first exponential expression contains the same term with *x *replaced by the constant, *c*_1_. The simple function of *x*, f(*x*) = *b*_2_*x*, in the second exponent can be replaced by any function of *x*, call it g(*x*), and the first exponent can be exchanged for g(*x*) with *x *replaced by a new constant, *c*_2_. The result will always be a transition between a zero-slope linear domain and g(*x*). For example, we could insert *y*_II _as g(*x*), and subtract the resulting term from *y*_II _itself:



With *c*_2 _and *d*_2 _appropriately adjusted, we thus create a second transition zone right of the first one (Fig. 2; *y*_III_). For *x *values significantly greater than *x *at the position of this second transition, this results in a third linear domain with zero slope.

At *x *= 0, equation 6 becomes:



where, for simplification, we define

*L *= ln (exp [*b*_2 _*c*_1 _*d*_1_] + exp [*b*_2 _*c*_2 _*d*_1_])     (8)

By definition, velocity equals zero at the point of reference (conventionally the root apex, *i.e. x *= 0 in our model), and we would like to see this feature in our function. Therefore we subtract *y*_III,0 _from y_III_:

*y*_IV _= *y*_III _- *y*_III,0 _    (9)

This function passes through the origin and asymptotically approaches a constant maximal value after the second transition zone (Fig. 2). In contrast to most classical growth equations, there is no parameter in our function which directly corresponds to this asymptote; it rather is determined indirectly by the geometry of the curve which results from the cooperation of all of the function's coefficients. The coefficients control the graph's shape in specific ways: *b*'s determine the slopes of the linear domains, which are *b*_1 _(first linear domain) and *b*_1 _+ *b*_2 _(second linear domain). The *c*'s are the *x *values at the positions of the first and second transition zone (*c*_1 _and *c*_2_, respectively), while *d*'s define the width of the transition zones (the greater *d*, the narrower the transition zone). When equation 9 is used to describe root growth zones, negative values of any *b*'s and *c*'s will be meaningless; similarly, *d*'s will be positive and *c*_2 _> *c*_1 _will always hold. When fitting the function to experimental data, it is advisable to implement these restrictions of possible coefficient values in the automated fitting algorithm to avoid unexpected results.

The complete function can be shifted along the y-axis by adding a constant *q*:

*y*_V _= *y*_IV _+ *q *    (10)

When fitting datasets which do not cover the region close to *x *= 0 (*i.e*. the root apex), this may be useful although it formally is a violation of the assumption that velocity is zero at *x *= 0.

### Derivatives of the step-stool function

The derivative of *y*_IV_, equalling that of *y*_V_, is:



where



and



This derivative gives us the profile of elemental growth rate along the growth zone. The second derivative of *y*_IV _also is helpful as it facilitates the identification of stationary points in the growth rate profile such as the position of its maximum:



## Results

**Figure 3:**
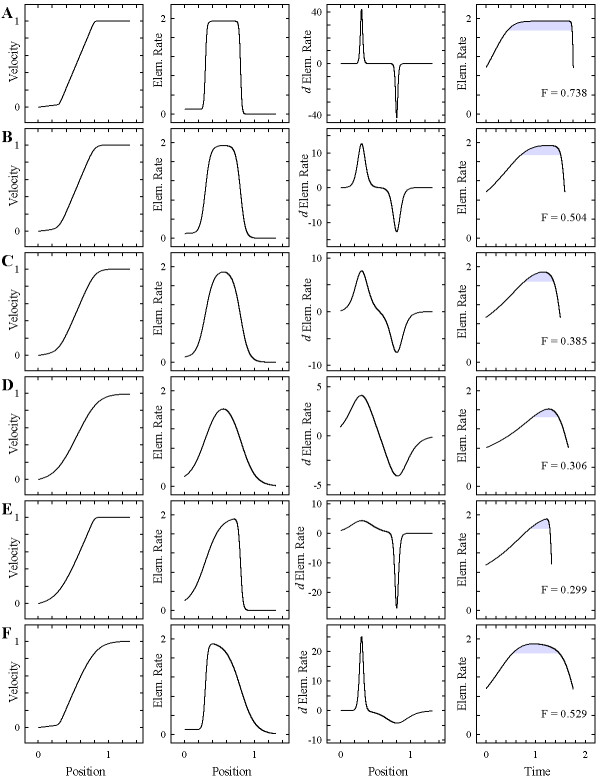
**Sample profiles demonstrating the flexibility of the step-stool model**. Growth zone models of increasing sigmoidal character (strongly bi-linear, A, to strongly sigmoidal, D), and two asymmetric examples with pronounced right (E) and left (F) skew. From left to right, columns show profiles of velocity, elemental elongation rates (first derivatives of the velocity profiles), second derivatives of the velocity profiles, and time-courses of elemental elongation rate, with time = 0 taken at the position where the rate reaches 50% of its maximal value. Grey shading highlights the period during which the organ element grows at >90% of its maximal elemental elongation rate (parameter F; see text for details). All models: *b*_1 _= 0.1, *b*_2 _= 1.84, *c*_1 _= 0.3, and *c*_2 _= 0.8. A: *d*_1 _= *d*_2 _= 50; B: *d*_1 _= *d*_2 _= 15; C: *d*_1 _= *d*_2 _= 9; D: *d*_1 _= *d*_2 _= 5; E: *d*_1 _= 5, *d*_2 _= 30; F: *d*_1 _= 30, *d*_2 _= 5.

First, we explored the flexibility of the step-stool function, *y*_IV_, and found that it could model any desired stage in the transition from bi-linear to sigmoidal growth profiles (Fig. 3A–D). As illustrated in Fig. 1, one of the biologically relevant differences between sigmoidal and bi-linear velocity profiles is the length of the period in which growing cells remain at their maximal growth rate. To quantify this parameter, we computed the time-course of elemental growth rate and expressed the flatness, F, of the curve as the ratio of the periods in which the root elements grew at greater than 90% and at greater than 50% of their maximal rate. As expected, flatness decreased with increasing sigmoidal character of the profile (Fig. 3F; is shown to the right).

Our function also could produce asymmetric profiles (Fig. 3E, F). The ability to describe asymmetric sigmoidal profiles is the hallmark of the Richards-function, a classical growth equation with four coefficients [27], which added flexibility to the so-called functional approach of growth analysis [28]. Figure 3E, F illustrates that our function achieved the aim of mimicking flexible growth equations such as Richards'.

**Figure 4:**
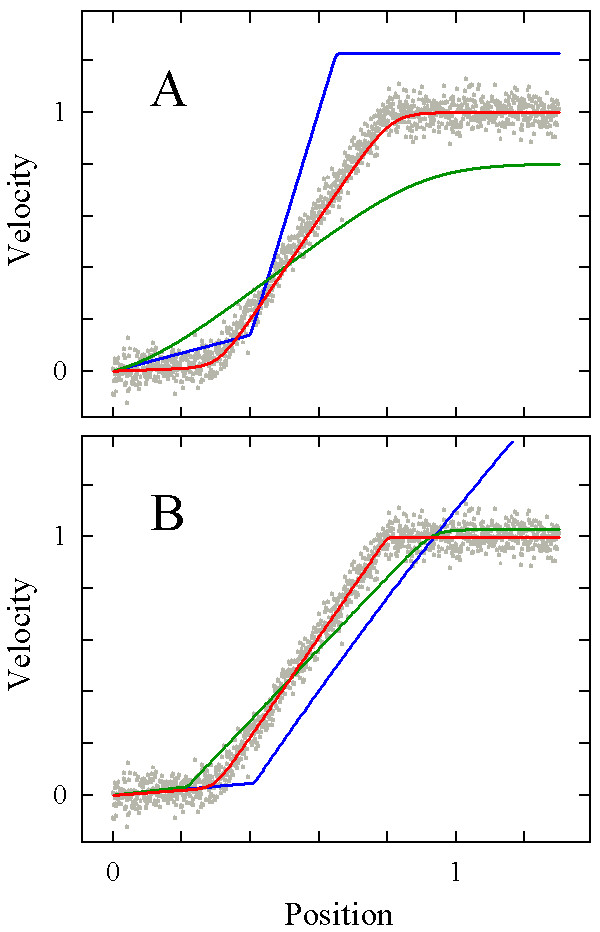
**Effects of initial values of coefficients on automated curve fitting**. A 10% level of Gaussian noise was added to a bi-linear model velocity profile to create a "Monte-Carlo-dataset" (gray dots, n = 1301; coefficients are *b*_1 _= 0.1, *b*_2 _= 1.84, *c*_1 _= 0.3, *c*_2 _= 0.8, *d*_1 _= *d*_2 _= 50), and the step-stool equation was fitted using three different sets of coefficient values to initiate the fitting algorithm. The graphs of three of these sets are shown in (A); the blue and the green curves are deliberately inappropriate, whereas the red one represents a "smart guess". The result of automated curve fitting starting from the initial values depicted in (A) is shown in (B) in corresponding colours. In the cases of poor initial value choice, the fitting algorithm became stuck at unacceptable solutions; in contrast, the "smart guess" provided a satisfying result.

**Figure 5:**
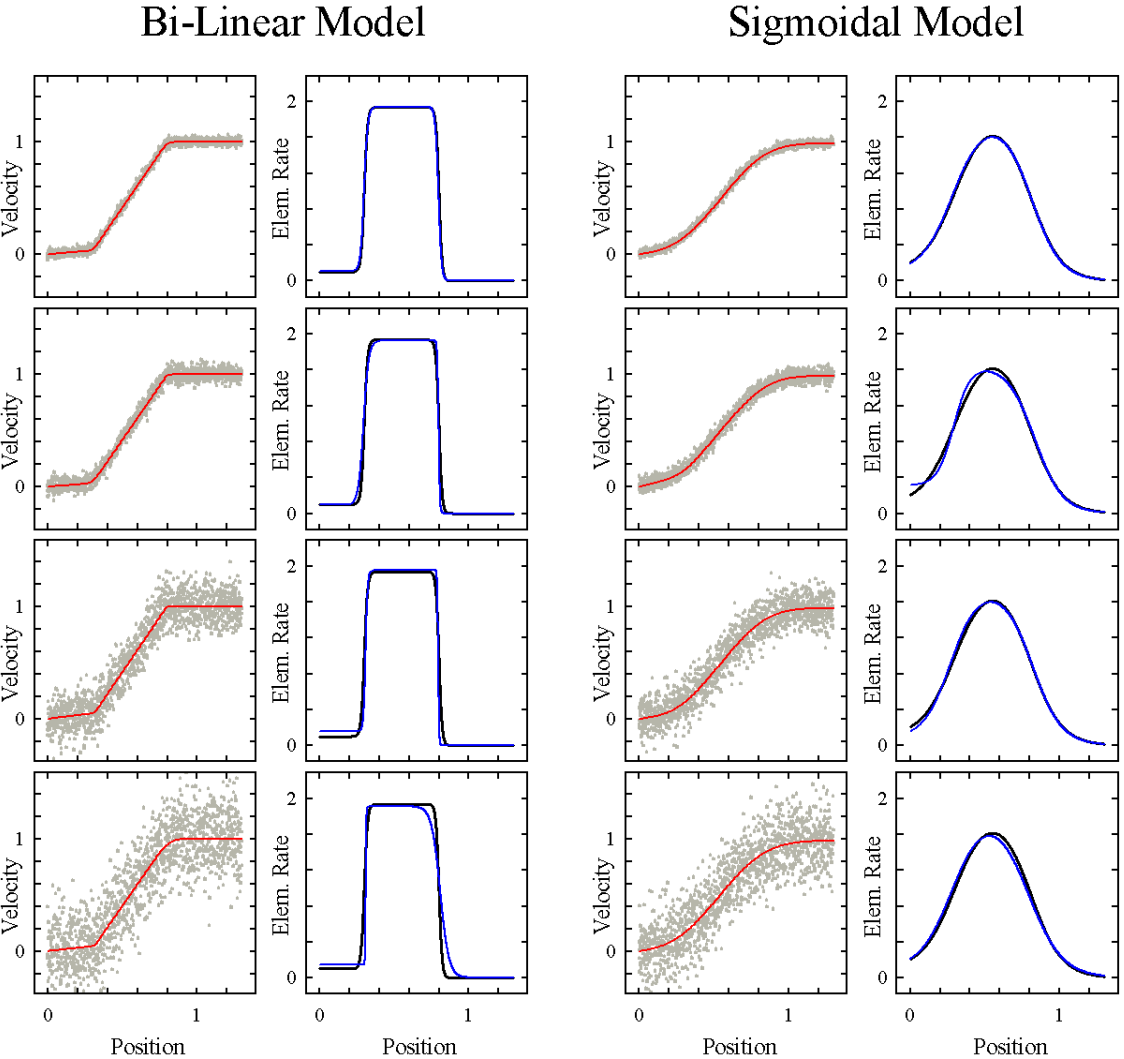
**Effects of noise on fitting the step-stool function **Increasing levels of Gaussian noise (from top to bottom) were added to a strongly bi-linear (left; *b*_1 _= 0.1, *b*_2 _= 1.84, *c*_1 _= 0.3, *c*_2 _= 0.8, *d*_1 _= *d*_2 _= 50) and a strongly sigmoidal (right; *b*_1 _= 0.1, *b*_2 _= 1.84, *c*_1 _= 0.3, *c*_2 _= 0.8, *d*_1 _= *d*_2 _= 5) model velocity profile to create "Monte-Carlo-datasets" (gray dots; n = 1301). A person unaware of the nature of this study produced "smart guesses" of coefficient values for the 50% noise level versions of both models (bottom), which were used to initiate the fitting for all of the noise levels; the fitted curves are shown in red. Derivatives (elemental elongation rate profiles) are given on the right of the corresponding velocity profile. Fitted profiles (blue) are practically identical with those expected (black).

Having established a step-stool function with promising features, we tested its behavior in automated curve fitting procedures. To this end, we created Monte-Carlo datasets [29]: various levels of Gaussian noise were added to several model velocity profiles, such as those shown in Figure 3, with a number of data points that was comparable to that of our empirical datasets. Then, the function was fitted using the Marquardt-Levenberg algorithm included in the non-linear regression tool of SigmaPlot, a scientific graphics and data analysis package widely distributed among biologists. Briefly, this algorithm searches for coefficient values for a best-fit of a given function as defined by the least-sum-of-squares criterion. The search procedure is an iterative improve-by-guess-and-try process which does not establish *the *correct result, but rather provides an estimate of an acceptable solution. The significance of the estimated coefficients and their mutual dependences need to be assessed by statistical analyses of the fitting results, which are provided automatically by modern software packages such as the one used here. Particularly in the case of complex, versatile functions, the fitting algorithm is sensitive to the initial coefficient values from which it starts, and a smart guess of these values is an essential step [30]. As expected, the step-stool function was sensitive in this respect (Fig. 4). However, when reasonable coefficient values had been established by an initial round of manual curve fitting (see Additional File 1 for details), the results of the subsequent automated fitting procedure were reproducible, robust against small changes in coefficient initial values, and insensitive to Gaussian noise in the datasets, for all bi-linear and sigmoidal velocity profiles examined (Fig. 5 gives two examples).

**Figure 6:**
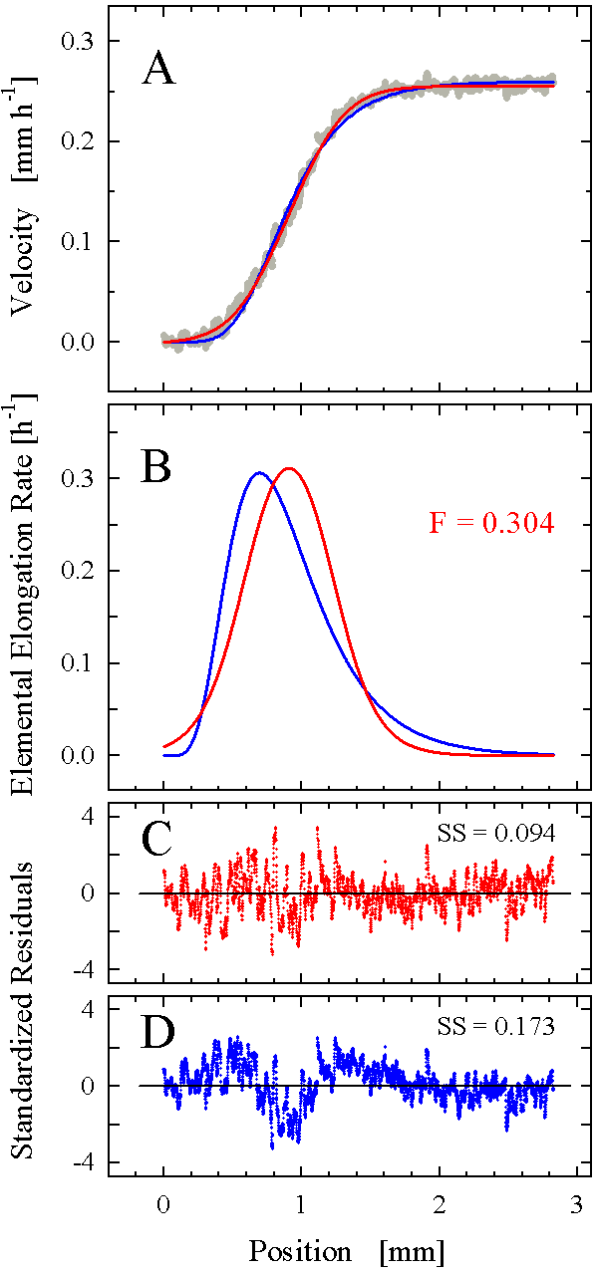
**Analysis of a velocity field from a *G. elegans *root**. (A) A 5-parameter version of the step-stool function lacking paremeters *b*_1 _and *q *(red) and the 4-parameter Richards function (blue) were fitted to the experimental velocity data (gray dots; n = 3678). (B) Derivatives of the velocity curves (elemental elongation rate profiles). The flatness, F, of the time-course of elemental elongation rate was determined for the step-stool model. (C) Standardized residuals for the step-stool model and (D) for the Richards model. The value of the primary criterion of goodness-of-fit, the sum of squared residuals (SS), is indicated in (C) and (D).

**Figure 7:**
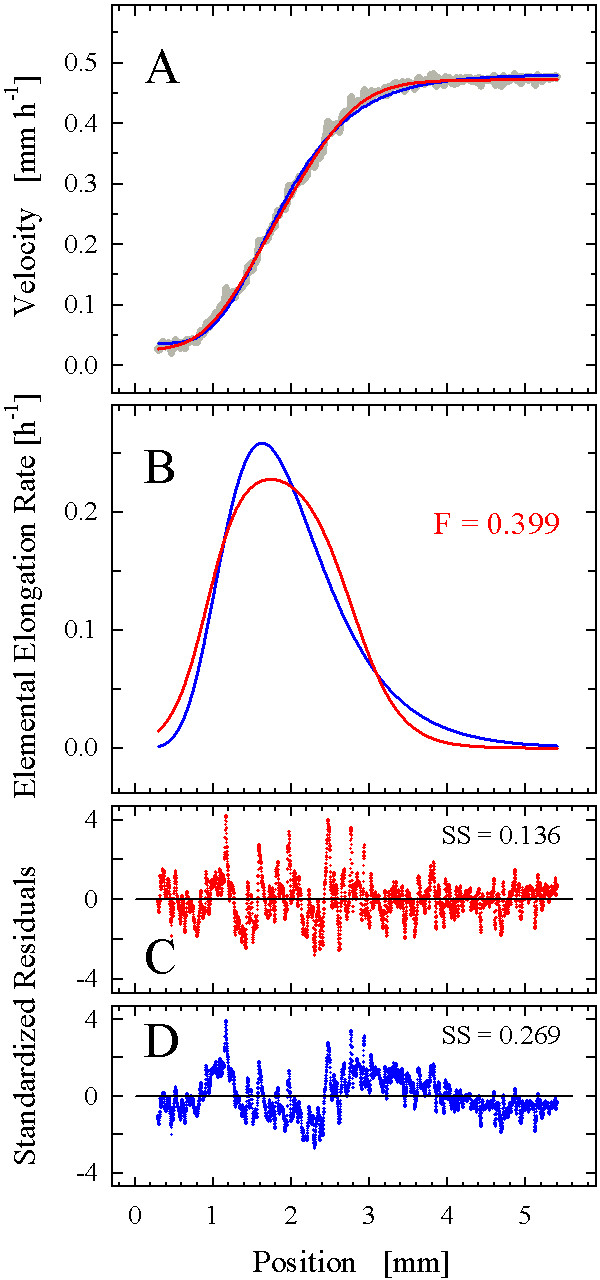
**Analysis of a velocity field from an *Au. saxatilis *root**. (A) A 6-parameter step-stool function including the parameter *q *but not *b*_1 _(red) and the 5-parameter version of the Richards function (blue) were fitted to the velocity data (gray dots; n = 3366). For details of (B), (C), and (D), see Fig. 6.

**Figure 8:**
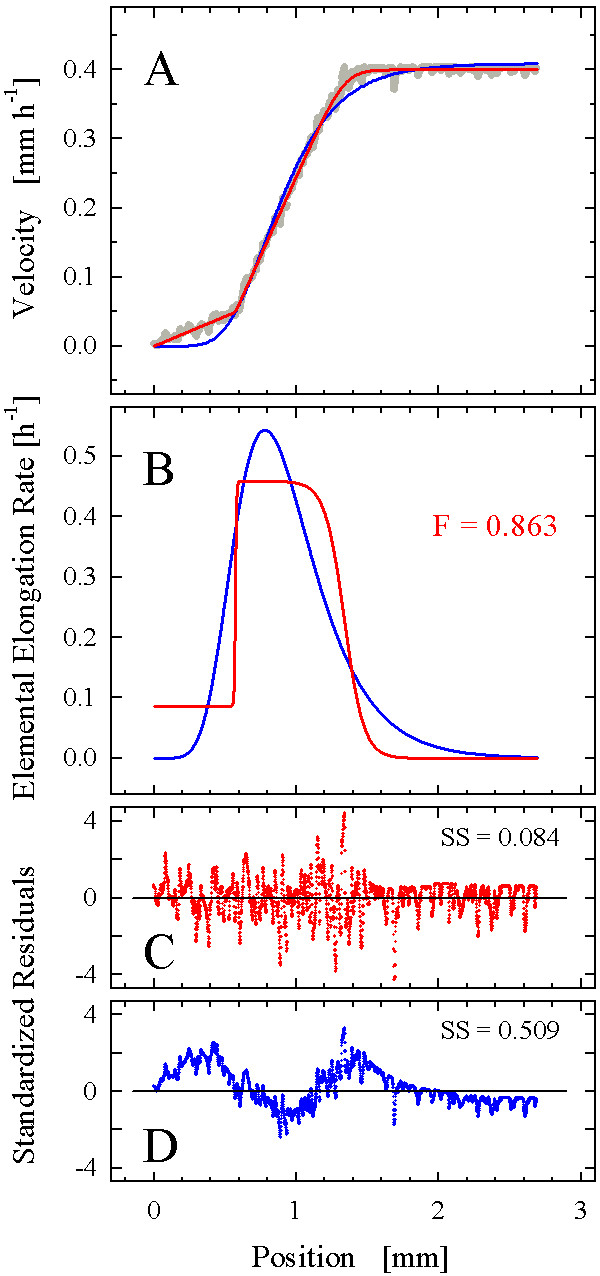
**Analysis of a velocity field from an *A. thaliana *root**. (A) A 6-parameter step-stool function lacking the parameter *q *(red) and the 4-parameter Richards function (blue) were fitted to the velocity data (gray dots; n = 2729). For further details, see Fig. 6.

On this basis, we proceeded to test the function on real root growth data. We selected three datasets produced by the high-resolution methodology recently introduced [12]; visual inspection suggested that these datasets possessed sigmoidal-like (*Gypsophila elegans*; Fig. 6), intermediate (*Aurinia saxatilis*; Fig. 7), and strong bi-linear (*Arabidopsis thaliana*; Fig. 8) characteristics. Fitting of the step-stool function invariably started with a manual adjustment of the coefficients to provide a "smart guess" of initial values for the subsequent automated fitting procedure. As the fitting of the step-stool function was intended to provide an independent criterion to decide whether a given dataset had more or less sigmoidal properties, we routinely included the following check for reliability of the fitting results. As the samples in Fig. 3A to 3D demonstrate, the degree of step-stool-likeness of the derivative of a fitted curve depends mostly on the coefficients *d*, which determine the abruptness of the transitions; the greater *d*_1 _and *d*_2_, the more abrupt the transitions and the less sigmoid-like the curve. To see whether the degree of sigmoid likeness indicated by the automated fit was robust, we repeated the fitting procedure twice, starting with initial values of the coefficients *d *either doubled or halved. In all our examples (Figs. 6, 7, 8), the differences between the fitting results were insignificant, indicating that the curves obtained represented the best fit of the step-stool function to the datasets.

Inspection of the dataset obtained from a *G. elegans *root (n = 3678) revealed a pronounced sigmoidal character; fitting of the step-stool function supported this conclusion (Fig. 6A; the derivative of the function fitted is shown in Fig. 6B). Because the dataset showed no indication of a non-zero slope for the linear domain close to the root apex, a truncated version of the step-stool function (*y*_IV_, equation 9) was used in which *b*_1_, the coefficient controlling the slope of the first linear domain, was set to 0. Thus, the fitted function had only five coefficients instead of the six of the complete version. To test for the appropriateness of our decision, we also fitted the complete version of the function. The resulting curve was practically the same as was the sum of squared residuals, with the estimated *b*_1 _having a value close to 0 and an enormous variance, indicating that *b*_1 _had virtually no effect on the goodness-of-fit (all other coefficients were significant and non-correlated). Thus, the fitting statistics confirmed that there was no justification to include the sixth coefficient, *b*_1_, in the fitted function for this data set.

To test the accuracy of the fit, we used Durban-Watson statistics (a test for correlation between residuals [31]) and the Levene median test for the homogeneity of variances of residuals [32], as implemented by the software package employed. The fit of the step-stool function failed in these tests, which could be explained by the presence of significant trends in the dataset that were not accounted for by the fitted function. To discover such trends, we examined a standardized residual plot (Fig. 6C). Residuals are the differences between the predicted and measured values; standardization divides them by the standard deviation of the particular set of residuals [33]. The plot showed that the residuals were distributed around the expected values in a non-stochastic manner; oscillatory deviations of various wavelengths from the predicted value were evident. This qualitative observation can be quantitatively assessed by a so-called runs analysis; here, a run is defined as a series of observations consisting of at least one measurement, in which all observations are either above or below the predicted values. For datasets comprising 3678 observations and fit to the correct model, residuals distributed stochastically would be expected to show 1840 runs (standard deviation = 30) [31]. However, the fit of the step-stool function in Fig. 6A produced only 181 runs, confirming the significance of the trends observed in the residual plot (Fig. 6C).

For comparison, we fitted the Richards function to the dataset (blue curve in Fig. 6A) and also plotted its derivative (Fig. 6B) and distribution of residuals (Fig. 6D). For this fit, the coefficient of determination was only slightly lower than for the step-stool function (r^2 ^= 0.995 compared to r^2 ^= 0.997). In contrast, the sum of squared residuals, the primary measure of goodness-of-fit used by the fitting algorithm, was almost doubled (0.173 compared to 0.094). Intriguingly, the estimate of the location of the maximal elemental elongation rate differed notably between the Richards and the step-stool function, because the profile defined by the Richards fit was left-skewed (Fig. 6B). Comparison of the standardized residual plots (Fig. 6C, D,) shows that a long-wavelength deviation from the measured values was more pronounced in the fitted Richards function, especially around the position of maximal growth rate. We conclude that the step-stool function, while not able to describe all minor trends present in this high-resolution dataset, provides a more accurate description of the data than does the Richards function.

For this data set, we were surprised by the inferior fits from the Richards function, given that it has a pronounced sigmoidal character and therefore falls within the purview of classical, sigmoidal growth functions such as Richards'. For the step-stool function, the fitting result statistics indicated highly significant contributions to the prediction of the independent variable by all of the five coefficients of the truncated function used. This finding justified the inclusion of a fifth coefficient (as compared to the Richards function, which has four) on statistical grounds; we further verified the conclusion by comparing the step-stool and Richards models by the corrected Akaike's information criterion (AIC_C_; [30]). This information theory-based criterion provides information on whether an improvement of goodness-of-fit due to the inclusion of additional parameters is significant. This stringent test is preferable to the popular F-test, particularly when comparing non-nested models, as done here [30] ([34] gives an in-depth discussion of the information-theoretical basis; for a more condensed introduction, see [35]). The AIC_C _score (equation 17) of the step-stool model was smaller than that of the Richards function (-38881 compared to -36640; ΔAIC_C _= 2241), confirming that the five-parameter step-stool-fit provided a better description of this particular dataset than the four-parameter Richards fit. To appreciate the robustness of this conclusion, note that a ΔAIC_C _of 4.6 implies that the model with the lower score is 10 times more likely to be true than its competitor [[30]; equation [18]]: with a ΔAIC_C _of 2241, the factor by which the step-stool model is more likely than the Richards function to be a correct description of this dataset is 10^487^!

The second dataset, this time from an *Au. saxatilis *root (n = 3366), did not extend to position *x *= 0 (Fig. 7A), exemplifying a situation that frequently occurs because the collection of reliable data from the root meristem where velocities approach zero is difficult. Moreover, the data available seemed to include an initial, zero-slope linear domain. Therefore, we fitted a truncated version of the step-stool function lacking the coefficient *b*_1 _as in the previous example, but including the constant, *q*, to enable non-zero values of the function at position *x *= 0 (equation 10). The fitted curve described the data well (Fig. 7A), and the analysis of variances of the estimated coefficient values showed that all six coefficients, including *q*, made significant contributions to the prediction of the dependent variable.

The shape of the fitted curve resembled that seen in the previous example, but the graph of its derivative appeared wider and flatter (Fig. 7B). As a consequence, the flatness value was increased to almost 0.4, indicating that in this root, cells grew near their maximal growth rate for a relatively longer proportion of their phase of expansion. Again, there were non-stochastic components visible in the residual plot, showing up as long-wavelength and short-wavelength oscillatory deviations (Fig. 7C). The fit of a five-coefficient version of the Richards function (including a constant, *q*, as in the step-stool function; Fig. 7A) produced a coefficient of determination slightly lower than that of the step-stool function (0.997 compared to 0.999), but the sum of squared residuals was twice as high (0.269 compared to 0.136) and the non-stochastic oscillations in the residual plot were more pronounced (Fig. 7D). Thus, the step-stool function provided a more accurate description of the dataset than the classical sigmoidal model, which was confirmed by its lower AIC_C _score (-34038 as compared to -31745).

In the third example, an *A. thaliana *root, the bi-linear character of the velocity profile was unambiguously visible in the raw data plot (Fig. 8A; n = 2729). As expected, the superiority of the description provided by fitting the step-stool function (the complete six-coefficient version, equation 9) as compared to the four-parameter Richards function was obvious from the plots of the fitted velocity curves alone (Fig. 8A; coefficients of determination were 0.999 and 0.992, and sums of squared residuals were 0.084 and 0.509 for the step-stool and the Richards function, respectively). As expected, the AIC_C _scores for the step-stool and the Richards model (-28337 and -23424, respectively), as well as the tests of significance and independence, showed that the inclusion of two additional parameters in the step-stool fit was justified. The biphasic character was clearly expressed in the derivative of the step-stool function (Fig. 8B); the flatness value of 0.86 indicated that cells in this root expanded near their maximal growth rate for a substantial part of their elongation phase. Comparison of the residual plots (Fig. 8C, D) confirmed that the sigmoidal Richards function provided an inappropriate description of this dataset. However, as in the cases discussed before, the fit of the step-stool function failed to pass the Durban-Watson and Levene median tests, indicating non-stochastic factors in the distribution of residuals (Fig. 7C) and consequently, the existence of significant trends in the dataset unresolved by the step-stool fit.

## Discussion

Recent technical advances have facilitated the demonstration of velocity profiles along root growth zones that consist of two distinct, nearly linear domains rather than being sigmoidal [12]. In contrast to the sigmoidal models, which dominate text-books and previous research reports, bi-linear growth profiles imply that cells switch between two distinct expansion modes when going through their period of elongation (Fig. 1). Because of this implication, the distinction of bi-linear from sigmoidal growth zones is essential in kinematic growth studies. The step-stool function was created as a tool to characterize the shape of velocity profiles without imposing either a sigmoidal or a bi-linear character. Because we currently do not possess a physiological hypothesis from which to derive a mechanistic, quantitative model of the regulation of velocity across a growth zone, the coefficients in the step-stool model refer to readily observed features of the profile, including slope, transition position and abruptness, rather than to underlying physiological processes. Biological meaning enters our analyses via the geometry of the fitted curve, which ultimately reflects the characteristics of the time-courses of physiological parameters experienced by cells traversing the growth zone (Figs. 1, 3).

Some velocity profiles may be truly sigmoidal while others may be bi-linear. Insofar as the possible results of fitting our function include both alternative extremes, our approach is a multiple-working-hypotheses one, which conceptually complements conventional model testing based on statistical quantification of the goodness-of-fit (as exemplified by the comparative evaluations of the step-stool and the Richards function; for a general discussion of multiple-working-hypotheses approaches in biological modelling, see [29]). In this context, the increased number of parameters in the step-stool function as compared to conventional growth models such as the Richards function should not be viewed as merely a means of improving the goodness-of-fit. Rather, it is the cost of gaining an additional criterion for model choice by fitting an equation capable of describing two competing models equally well. Because there is no exclusive answer as to whether a given dataset is either sigmoidal or bi-linear, a pragmatic measure of "bi-linearity" has to be defined. As one possibility, we here introduced F, the flatness of the time-course of elemental growth rate.

Our analyses of noisy Monte-Carlo-datasets and three exemplary experimental profiles demonstrated the success of our approach: the step-stool function provided better fits in all cases than did the Richards function; noteworthily, the latter is superior to other sigmoid functions due to its flexibility and therefore is considered a standard in growth analysis [27,28,36]. The flexible application of the step-stool function, which includes "smart guessing" of initial parameter values as well as the addition or deletion of coefficients depending on the properties of a particular dataset, certainly requires some familiarity with the mathematical basis of non-linear curve fitting. However, this should not be a major obstacle, given the availability of powerful, user-friendly software and desktop computing power that was unthinkable of at the time the Richards function was introduced. In general, the flexibility of tailor-made modular functions created to assist the solution of specific analytical problems is an asset in cases in which no mechanistic hypothesis has been developed, and from which explanatory mathematical models with biologically meaningful interpretations of parameters could be derived.

The application of the step-stool function may not be limited to kinematic growth analysis, as the necessity to distinguish between continously and abruptly changing curves is a notorious source of problems in classical plant growth analysis [24] as well as in the analysis of procaryotic [37-39] and eucaryotic [39-41] cell expansion. Formally similar problems exist in the field of biochemical [42,43] and transport kinetics [44]. Moreover, we currently are applying extended, tri-linear versions of the step-stool function in the description of polyphasic stomatal movements and the spatio-temporal quantification of volume changes in contractile forisomes [45].

With respect to the kinematic analyses discussed in this study, the finding of a velocity profile that is unambiguously a sigmoid implies that under certain conditions cells may regulate elemental elongation rate rather smoothly as they traverse the growth zone. This possibility merits further study; however, the purpose of the present work is to test the behavior of the step-stool function, and it will be up to subsequent investigation to determine whether a sigmoid profile for *G. elegans *indeed represents root growth in that species.

The application of the step-stool function will also form the basis for further investigations into the causes of the non-stochastic trends evident in the residual plots (subfigures D in Figures 6, 7, 8). It cannot be excluded that such deviations may occur as artifacts in the high-resolution computational analysis applied to serial images of growing roots [12]. However, we consider it more likely that the oscillatory deviations reflect the behaviour of cells or cell groups that grow slightly faster or slower than the average growth curve suggests. This interpretation is substantiated by a previous report suggesting that local minima and maxima of growth rate travel along the growth zone similarly as cells do [46]. Future studies will aim to establish the nature of these local deviations of growth rate.

## Conclusion

We present here an empirical function, with six coefficients, that is able to fit one-dimensional velocity profiles regardless of whether they are bi-linear or sigmoidal. Furthermore, the values of the coefficients provide analytical estimates of the key parameters of the profile, including the local slope, the position where the change of slope is centered, and a parameter characterizing the abruptness of the change in slope. The function is appropriate for data sets with thousands of points, as generated by modern, digital methods of velocity estimation, and fitting the function is robust to Gaussian noise. Finally, we demonstrate examples of velocity profiles from real roots that are sigmoidal, bi-linear, and intermediate. We suggest that this function will facilitate kinematic analysis of growth and that our strategy for constructing the function may prove to be useful in general for quantitative biology.

## Methods

### Plant material and growth measurement

The plant material and growth measurement have been described in full previously [12]. Briefly, seedlings of *Arabidopsis thaliana *(L.) Heynh. Columbia background, *Aurinia saxatilis *(L.) Desv., and *Gypsophila elegans *Bieb. were grown on the surface of a nutrient-agar medium in vertical Petri dishes under continuous light. A plate was put on the stage of a horizontal microscope and a stack of nine images collected of the primary root with 10 seconds between images. A series of four to eight stacks, depending on the root, was captured, from the root tip to an area with mature root hairs. The velocity field was recovered from these image sequences by RootflowRT, software that recovers dense velocity fields for deformable motion based on principles of optical flow. Velocities are obtained for essentially each pixel in the image and the component parallel to the local tangent of the root's midline is averaged perpendicular to the midline to produce the one-dimensional velocity fields used here. Further details available in [12] and [47]. RootflowRT may be downloaded from [48].

### Curve fitting

All mathematical operations including the generation of model velocity fields, creation of Monte-Carlo datasets by adding Gaussian noise to these models, and numerical integration were performed using SigmaPlot (version 7.101, SPSS Inc., Chicago IL, USA) and TableCurve 2D (version 5.1, Systat Software Inc., Richmond CA, USA). Plotting of graphs and automated curve fitting was carried out with SigmaPlot; standard procedures implemented in this software were used to statistically analyze the fitting results. These tests included estimations of the standard errors, coefficients of variation, dependencies, t-statistics and P values of the parameters fitted, which were used to judge parameter significance and non-correlatedness. Special attention was paid to the residuals, which were tested routinely for correlation by Durbin-Watson statistics, normal distribution around the regression, and constant variance by the Levene median test.

The construction of the step-stool-function is described in detail in the Theory section. For comparison, the Richards function (Richards, 1959) was also fitted to experimentally determined velocity profiles (*a*, *b*, *c*, *d *are constants, *x *is the variable):

*y *= *a *(1 - exp [*b *- *cx*])^*d *^    (15)

It should be noted that the graph of this function invariably intersects the ordinate at *a*(1 - exp [*b*])^*d*^; a fifth parameter has to be added if flexible intersections are required. The derivative of the Richards function is



To determine whether an improvement of the goodness-of-fit due to the inclusion of additonal parameters in a model was significant, we calculated the corrected Akaike's information criterion (AIC_C_) according to [30]:



where *n *is the number of datapoints, *k *is the number of parameters fitted plus one, and SS is the sum of squared residuals. The difference of the AIC_C _scores (ΔAIC_C_) of two competing models is a function of the relative likelihood of each of them to be a true description of the particular dataset to which they have been fitted; the model with the lower AIC_C _is more likely to be true by a factor known as the evidence ratio:



## Competing interests

The author(s) declare that they have no competing interests.

## Authors' contributions

T.I.B. contributed the experimental growth data, W.S.P. formulated the step-stool equation and performed the statistical analyses. Both authors conceived of this study while discussing their previous work and produced the manuscript cooperatively.

## Supplementary Material

Additional File 1 **PETERS_BASKIN_Manual_Fit**. Template file for manual fitting of the step-stool function. See sheet 1 for instructions on how to operate the manual curve-fitting template on sheet 2. Click here for file
